# The Role of Telomerase and Telomeres in Interstitial Lung Diseases: From Molecules to Clinical Implications

**DOI:** 10.3390/ijms20122996

**Published:** 2019-06-19

**Authors:** Nissim Arish, Dmytro Petukhov, Shulamit B. Wallach-Dayan

**Affiliations:** 1 Pulmonary Institute, Shaare Zedek Medical Center, P.O. Box 3235, 12 Shmuel Bait St., 9103102 Jerusalem, Israel; nissim.arish@gmail.com; 2 Lung Cellular and Molecular Biology Laboratory, Institute of Pulmonary Medicine, Hadassah–Hebrew University Medical Center, P.O. Box 12000, Qiryat Hadassah, 91120 Jerusalem, Israel; petukhov@hadassah.org.il

**Keywords:** telomere, interstitial lung disease, idiopathic pulmonary fibrosis, telomerase, shelterin, long noncoding RNA

## Abstract

Telomeres are distal chromosome regions associated with specific protein complexes that protect the chromosome against degradation and aberrations. Telomere maintenance capacity is an essential indication of healthy cell populations, and telomere damage is observed in processes such as malignant transformation, apoptosis, or cell senescence. At a cellular level, telomere damage may result from genotoxic stress, decreased activity of telomerase enzyme complex, dysfunction of shelterin proteins, or changes in expression of telomere-associated RNA such as TERRA. Clinical evidence suggests that mutation of telomerase genes (*Tert/Terc*) are associated with increased risk of congenital as well as age-related diseases (e.g., pneumonitis, idiopathic pulmonary fibrosis (IPF), dyskeratosis congenita, emphysema, nonspecific interstitial pneumonia, etc.). Thus, telomere length and maintenance can serve as an important prognostic factor as well as a potential target for new strategies of treatment for interstitial lung diseases (ILDs) and associated pulmonary pathologies.

## 1. Introduction

Telomeres are nucleoprotein complexes that assemble at the end of chromosomes. They comprise tandem repeats of a short DNA sequence, such as TTAGGG for humans and other vertebrates [1]. Telomere length is under tight control because the telomere protects the chromosome from degradation, erroneous recombination and fusion [2]. Telomere maintenance and length control are obtained by the function of the telomerase enzyme complex, shelterin proteins [2] and telomere-associated RNAs [3].

Interstitial lung disease (ILD) is a diverse group of different pathological conditions that mainly affect the pulmonary parenchyma [4]. Idiopathic pulmonary fibrosis (IPF) is a progressive fibrotic lung disease of unknown etiology and is the most common form of ILD. The term IPF is reserved for patients with usual interstitial pneumonia (UIP) patterns of lung disease, which is characterized on chest CT scans by subpleural, basal-predominant reticular abnormalities, traction bronchiectasis and honeycombing, and histologically by patchy involvement of the lung parenchyma by fibrosis and micro honeycombing and the presence of fibroblast foci [5].

Most cases of IPF are sporadic; the occurrence of pulmonary fibrosis in multiple members of the same family, commonly referred to as familial pulmonary fibrosis (FPF), accounts for 2–20% of the overall cases of IPF, suggesting gene-environment interaction [6]. The diagnosis is per exclusion. It is defined as a form of UIP in the absence of known secondary causes, such as autoimmune diseases or known environmental exposures. The combined input of clinicians, radiologists and pathologists is recognized as being the most accurate way to diagnose the disease [5].

Smoking and aging are well-established risk factors for IPF. Various factors may be involved in the pathogenesis of ILD, such as chromosomal damage [7], DNA repair deficiencies [8] and epithelial-mesenchymal transition due to endoplasmic reticulum stress [9,10]. These factors may result in distinctive gene expression changes [11], which may serve as diagnostic markers [12], and in DNA damage under interstitial lung disease, specifically under pulmonary fibrosis. As telomeres form protective structures at the ends of chromosomes, it is imperative to understand their role in development of ILDs and to evaluate them as potential targets in antifibrotic therapy.

While the precise origin of IPF remains elusive, the disease probably results from repetitive alveolar injury coupled with dysfunctional alveolar wound healing mechanisms [13]. The disease progression often varies, but the prognosis is poor; median life expectancy is three years.

There is no cure for the disease, and referral to lung transplantation is recommended. Two FDA-approved anti-fibrotic drugs (Pirfenidone and Nintedanib) are known to hinder the disease progression by reducing the annual decline in forced vital capacity (FVC). Especially in early treatment [14,15].

## 2. Experimental ILDs

### 2.1. Telomerase-Deficiency and Spontaneous Development of Experimental ILD

The holoenzyme of telomerase consists of a catalytic subunit (telomerase reverse transcriptase (Tert)), regulatory subunits and an RNA cofactor (telomerase RNA component (Terc)) [16]. Telomerase function is critical to the maintenance of telomeres.

The role of telomerase activity and the significance of telomere length to the ILD susceptibility in experimental models remains a contested subject. Degryse et al. found that neither Tert^−/−^ nor *Terc*^−/−^ telomerase mutations are associated with the development of spontaneous fibrosis [17]. Lee et al. showed that under normoxia, telomerase-null mice (Terc^−/−^) show a compromised alveolar epithelial type 2 integrity after being inbred for only four to six generations, but do not develop spontaneous fibrosis [18].

This could be explained by the fact that telomerase deficiency in itself does not immediately lead to a decrease in the length of telomeres [19]. Instead, the telomere shortening is gradual through the consecutive generations [20] or accumulation of DNA damage [21]. The number of DNA breaks increases in each consecutive generation of telomerase-deficient mice [18], as detected, for example, by activation of the SAPK/JNK stress response [22].

### 2.2. Stress-Induced ILDs in the Context of Deficient Telomerase Function

Telomere shortening due to telomerase deficiency (Tert^−/−^), combined with inbred generations (four to six), increases the susceptibility of mice to stress-induced pulmonary fibrosis. Povedano et al. showed that the doses of bleomycin required to initiate lung fibrosis were significantly lower than in wild-type animals [23]. Liu et al. further demonstrated increased susceptibility of these mice to pulmonary fibrosis following exposure to liposaccharide [24]. Alder et al. demonstrated pulmonary emphysema in Terc^−/−^ mice following their exposure to smoke [25].

### 2.3. Experimental ILDs in the Context of Irregular Telomere Maintenance

**Figure 1:**
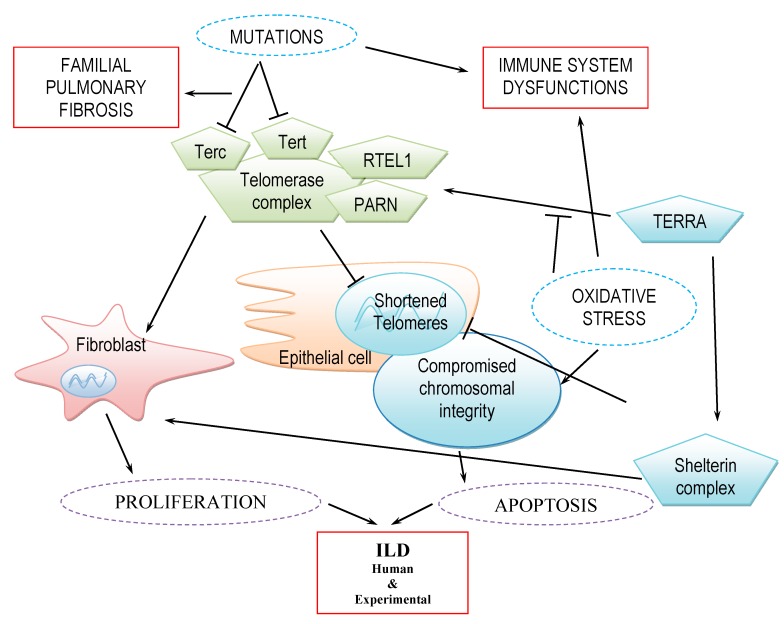
Principal factors involved in the regulation of telomere maintenance in the fibrotic lung. External or internal factors (such as mutations and oxidative stress, indicated by dashed blue ovals) may positively (pointed arrow) or negatively (block arrow) affect the telomere maintenance machinery through telomerase complex (green pentagon) or TERRA and shelterin complexes (blue pentagons), leading to structural and functional damage of telomeric and chromosomal DNA (solid blue ovals). The results of these disruptions depend on the affected cell type and vary from apoptosis in epithelial cells to cellular proliferation in fibroblasts (dashed violet ovals), thus causing imbalances in cell populations of the organ in question. In lungs, in particular, this may produce a proliferation of fibrotic tissue that manifests itself as an ILD, such as IPF or familial pulmonary fibrosis (red squares).

Telomere maintenance is supported not only via telomerase-mediated elongation but also by proteins (shelterin proteins) [2] and RNA molecules (long non-coding RNAs (lncRNAs)) [26] that physically act as a barrier against damaging agents and chromosome fusion (Figure 1). These protein and RNA molecules can also cooperate to enable telomerase activity per se [2].

Shelterin is a multi-protein complex consisting of telomeric repeat binding factors 1 and 2 (TRF1 and 2), TRF1-interacting protein 2 (Tin2), tripeptidyl peptidase 1 (TPP1), repressor-activator protein 1 (Rap1) and protection of telomeres protein 1 (POT1) (Figure 1). Shelterin proteins bind to telomeric DNA sequences and are critical to telomere length maintenance and telomere integrity [27]; changes in their expression are associated with telomere dysfunction such as shortening of the 3′ overhang or end-to-end chromosome fusions [28].

CST complex, which in humans consists of CTC1, STN1, and TEN1 proteins, is localized at the single-strand 3′ overhang and is involved in telomere end regulation [29]. It has been shown that under normal conditions CST complex restricts telomerase activity to one replication per cycle by interaction with TPP1 protein of the shelterin complex; however, this regulatory mechanism is disrupted under pathological conditions such as malignant transformation [30].

Shoeb et al. demonstrated that pulmonary fibrosis (modeled in rats by silica inhalation) is associated with disruption of TIN2-TPP1-POT1 shelterin complex leading to telomere damage [31]. In experiments on Trf2^Fl/Fl^ mice, Alder et al. determined that telomere dysfunction leads to alveolar stem cell failure via the P53-mediated pathway [32].

lncRNAs are RNA transcripts that do not serve as protein templates but are instead involved in epigenetic regulation together with other non-coding RNA subtypes, such as siRNAs [33]. Cao et al. have shown that the expression of a large number of lncRNAs is affected in pulmonary fibrosis [34]. Specifically, out of 568 differentially expressed lncRNAs, 210 were upregulated and 530 were downregulated. The most notably upregulated lncRNA in terms of fold increase and raw intensity were AJ005396 and S69206. In particular, increased expression of several lncRNAs was detected in fibrotic tissue and associated with renin-angiotensin signaling, the PPAR signaling pathway, the chemokine signaling pathway, adhesion molecule signaling, Jak/STAT signaling and other signaling pathways in bleomycin-induced lung fibrosis [34].

One lncRNA is telomeric repeat-containing RNA (TERRA), which is of high importance to telomere stability [2]. TERRA is known to bind to the shelterin complex in non-transformed cells, and to regulate telomerase replication activity [2]. Gao et al. further demonstrated increased expression of TERRA correlating with susceptibility to oxidative stress and apoptosis in alveolar epithelial type 2 cells of ILD patients and mice with bleomycin-induced fibrosis (Figure 1) [35]. Inactivation of TERRA improved cellular response in mice with bleomycin-induced fibrosis [35].

### 2.4. ILD Development and Telomere Length in Fibroblast vs. Epithelial Cells 

The population of cells involved should be regarded as a critical factor in research on telomerase function in pulmonary fibrosis. For instance, Liu et al. showed that in conditional-knockout murine models, mesenchymal-specific TERT inactivation leads to attenuation of bleomycin-induced pulmonary fibrosis [36].

Naikawadi et al. demonstrated that deletion of telomere cap shelterin gene *Trf1* in lung fibroblasts caused pulmonary edema, but not fibrosis, unlike deletion of *Trf1* in alveolar epithelial type 2 cells, which, with an increase in age, led to lung remodeling and spontaneous fibrosis [37]. Povedano et al. showed that Tert^−/−^ mice with shelterin *Trf1* deletion, in alveolar epithelial type 2 cells, develop pulmonary fibrosis due to severe telomere protein cap dysfunction [38].

Of note, these findings also indicate that telomere damage in alveolar epithelial type 2 cells is sufficient to cause spontaneous lung fibrosis in the elderly, and may be a major molecular defect causing IPF in humans [37]. In our studies, both *Tert* mRNA expression and activity decreased in murine type 2 lung epithelial cells under sufficient bleomycin exposure, compared with untreated cells, which led to increased apoptosis [39].

Concomitantly, Arish et al. [40] and Povedano et al. [23] demonstrated that specific telomerase activation in epithelial cells of mice with bleomycin-induced fibrosis might be a compensatory mechanism to alleviate lung epithelial damage [39]. Potential therapeutic agents acting through activation of telomerase function have been tested in murine models of experimental ILDs [41]. Notably, it was demonstrated by Le Saux et al. that activation of telomerase with the small molecule telomerase activator GRN510 leads to attenuation of fibrosis on mice with bleomycin-induced pulmonary fibrosis (as measured by reduced collagen deposit, diminished loss of lung function and protection of lung epithelial cells from cell senescence) [41].

## 3. Telomere and Telomerase in Human ILDs

The first clinical association between telomerase and lung disease was found in dyskeratosis congenita [42].

The characteristic features of dyskeratosis congenita are nail dystrophy, abnormal skin pigmentation and mucosal leukoplakia in the lungs. Bone marrow failure and pulmonary fibrosis are the primary causes of death. There are multiple patterns of inheritance, and the autosomal dominant form has a later age of onset. The X-linked form is due to mutations in the gene-encoding dyskerin, which is involved in processing the template RNA component of telomerase (Terc). Individuals with X-linked dyskeratosis congenita have low levels of Terc and, therefore, less telomerase activity [42].

Mutations in telomerase complex genes such as *Tert*, *Terc*, *RTEL1*, *PARN* or *DKC1* are found to be associated with a higher risk of pulmonary fibrosis (Figure 1). Indeed, the two genes, *RTEL1* and *PARN*, have been associated with shortened telomere lengths and familial pulmonary fibrosis (FPF) [43]. These genes are related to the premature shortening of telomeres in the peripheral blood and lungs, assessed as short telomere syndrome. Nevertheless, not all individuals with relevant mutations have short telomeres or develop ILD [44]. *RTEL1* mutation has been evidenced in 5%–9% of cases of familial pulmonary fibrosis; patients with ILD and *RTEL1* mutations present with various pulmonary and extra-pulmonary phenotypes, including sarcoidosis and RA-ILD. Hematological features are rare [45]. Heterozygous mutations of the protein component of telomerase (Tert) are the most frequently evidenced mutations observed in about 15% of affected families, while heterozygous mutations of the RNA component of the enzyme (Terc) are rarer.

It is believed that the loss of function of the telomerase complex may influence the turnover and healing of alveolar epithelial cells after a damaging stimulus, thus triggering IPF. Moreover, mutations in Tert/Terc are also associated with extra-pulmonary abnormalities, including premature hair greying, bone marrow failure and liver cirrhosis. The phenotype may be heterogeneous, even in patients with the same mutation [46].

It is important to emphasize that IPF is an age-related disease—it is rare to find IPF in young patients under 50 years old. Therefore, the connection to the telomerase gene is inferred. IPF is a prototype of telomere-mediated disease in slow turnover tissue such as the lungs, liver and pancreas. In these slow turnover tissues, telomere dysfunction disturbs organ homeostasis because of cumulative hits in long-lived cells and eventually culminates in what appears as an irreversible adult-onset disease [47]. Armanios et al. found that 8% out of 72 probands of familial IPF had a heterozygous mutation in *hTert* or *hTR*. They did not identify any symptoms of dyskeratosis congenita in these patients [48]. Another important finding was that asymptomatic young carriers of the mutation tended to have shorter telomeres than non-carriers. They hypothesized that telomerase length may serve as a surrogate marker for the identification of patients at a higher risk of carrying mutant telomerase genes, and may facilitate early detection of the disease. El-Chemaly et al. [49] followed two patients with preclinical familial pulmonary fibrosis with a mutation in the Tert gene for 27 years. One patient developed severe disease and was treated with oxygen, and the other remained asymptomatic but was found to develop pulmonary fibrosis on a high-resolution CT chest scan. The mutations are not only in the familial IPF; one mutation in *Tert* was found in a subject with the sporadic form of the disease who had no family history of IPF [50].

Short telomeres are a risk factor for IPF [51]. Cronikhile et al. measured the length of telomeric DNA isolated from circulating leukocytes of healthy control subjects and subjects with pulmonary fibrosis. Their findings showed a significantly higher proportion of probands with familial pulmonary fibrosis (24%), while sporadic case subjects (23%) had short telomere lengths that could not be explained by coding mutations in telomerase [52]. In a Spanish observational prospective study, the authors looked at predictive factors and the prognostic effects of telomere shortening in pulmonary fibrosis. They found that family aggregation, an age of <60 years and the presence of nonspecific immunological or hematological abnormalities were associated with a higher probability of telomere shortening and presented a poorer prognosis [53].

Telomeres were found to be significantly shortened in all forms of ILDs, including NSIP, cryptogenic organizing pneumonia (COP), hypersensitivity pneumonitis (HP), and others. However, it has been shown that telomeres in IPF were significantly shorter than in other forms of ILDs [4].

Smoking is a significant risk factor for developing IPF. One study showed that both current and former smokers had shorter telomeres than did age-matched nonsmokers [54]. In addition, there is some evidence that alveolar epithelium telomeres in smokers are shorter than those of the alveolar epithelium in nonsmokers. It is therefore possible that somatic telomere shortening, caused by conditions that increase cell turnover (e.g., smoking), could contribute to fibrosis [55]. Smoker patients with a *Tert* mutation can develop not only ILD but also emphysema.

In two independent cohorts, Stanley et al. found 3 of 292 severe COPD cases carried deleterious mutations in Tert (1%). This prevalence is comparable to the frequency of alpha-1 antitrypsin deficiency (the most known mutation in young emphysema patients) documented in this population. The Tert mutations compromised telomerase catalytic activity, and mutation carriers had short telomeres. Telomerase mutation carriers with emphysema were predominantly female and had an increased incidence of pneumothorax. In families, emphysema showed an autosomal dominant inheritance pattern, along with pulmonary fibrosis and other telomere syndrome features, but it manifested only in smokers [56]. This observation can explain the combined pulmonary fibrosis emphysema syndrome (CPFE).

## 4. Clinical Implications

Mutations in telomerase genes and short telomeres influence the course of the disease and tend to have a worse prognosis [57]. One observational cohort study looked at 77 subjects, 46% of whom met the diagnostic criteria for IPF with the rest being other forms of interstitial pneumonia. The results suggest a more rapid decline in FVC (300 mL per year) in IPF carriers with Tert mutations. Another interesting observation was that there were no significant differences in time to death or transplant for patients across gene mutation groups or patients diagnosed with IPF versus non-IPF. This finding contradicts the clinical experience of IPF as more progressive and having a worse prognosis in comparison with other forms of ILDs [58]. It is possible that genetic mutation in telomerase is more critical to disease progression than the etiology or the histology pattern of an ILD. Alternatively, it can be a marker for accelerated disease in other forms of ILDs. Table 1 lists telomere-associated mutations known to aggravate ILD-associated pathologies.

Today, immunosuppressive medication and prednisone are contraindicated in IPF patients. The PANTHER trial demonstrated increased risks of death and hospitalization in patients with idiopathic pulmonary fibrosis who were treated with a combination of prednisone, azathioprine and N acetyl cysteine as compared with a placebo [62]. Newton et al. described a strong correlation between the presence of reduced telomere length below the 10th percentile and the harmful effect of immunosuppressive medication in patients from the PANTHER clinical trial [63]. The effects of immunosuppressive therapy in other forms of ILDs with a telomerase-related gene mutation are as yet unknown.

Shortened telomeres in patients with IPF who carry a Tert mutation may respond less to treatment with pirfenidone. Therefore, the role of antifibrotic therapy in these patients is still unclear, and it should be managed with caution once there is a risk of liver injury associated [64].

Recently, in a prospective study, danazol, a synthetic androgen, was shown to increase telomere length, and seemed to stabilize DLCO among seven enrolled patients with pulmonary function tests available before danazol administration in telomerase mutation carriers. Pulmonary fibrosis scores based on CT were stable during the two years of treatment in all patients except one, who died from acute exacerbation of pulmonary failure [65].

As mentioned before, lung transplantation is the only definitive treatment for patients with IPF. A few small cohort studies suggest that patients with a mutation in the telomerase gene have more complications and a worse outcome after lung transplantation [66,67,68]. These observations were confirmed in a prospective study including 262 IPF lung recipients. In this cohort, 31 patients (11.8%) had variants in *Tert, RTEL1 or PARN*, whereas 231 (88.2%) did not. It was shown that IPF patients with a mutation in telomerase-associated genes had a significantly higher risk of death and chronic lung allograft dysfunction (CLAD) than patients without these variants. There was no difference in acute rejection or grade 3 primary graft dysfunction between the two groups [69].

A recent study in lung-transplanted patients showed a high prevalence of relapsing CMV viremia in IPF in comparison with non-IPF patients. IPF-transplanted patients who had short telomeres had the highest risk of CMV complications (*p* < 0.01) including relapsing-viremia episodes, end-organ disease and CMV resistance to therapy, as well as shorter time to viremia versus age-matched non-IPF control subjects (*p* < 0.001) [70].

Significant advancements made in the field of understanding the involvement of the telomeres in the pathogenesis of ILD leave open many possibilities for diagnostic and therapeutic attention [71]. This is especially important since IPF remains incurable (short of lung transplantation), and telomerase or other telomere-targeted approaches may offer new clinical options [23]. Short telomere screening is proposed as a useful tool in preferential selection for a lung transplant in ILD, since patients with short telomeres have poorer lung-transplant-free survival times [72]. For example, it has been determined through patient cohorts with various ILDs that decreased telomere length is associated with faster lung function decline and worse transplant-free survival, thus confirming its credence as a prognostic tool [73].

## 5. Perspectives for Further Studies

Significant advances have been made in the field of telomere research and specifically on the role of telomere maintenance in ILDs. The regulation of telomerase activity in lung tissue cells under various conditions (normal as well as pathology) has been elucidated in numerous studies [39,40,41,74]. The participation of shelterin proteins in that regulation, as well as their boarder role in telomere maintenance and telomere shortening under pulmonary pathology, has been investigated to a degree [31,44]. Also, recent advancements in understandings of the epigenetic mechanisms underlying participation of lncRNAs in telomere maintenance have made a significant impact on studies of the involvement of lncRNA, and TERRA in particular, in the pathogenesis of ILDs [2,33,34,35]. Many of these advancements are finding their way into clinical applications.

Nevertheless, many details remain unclear in the complex field of the role telomeres play in the pathogenesis of ILDs. For instance, although some factors such as GRN510 [41] or TERRA [35] have been named as potential regulators of telomerase activity in IPF, the particularities of their interactions are as yet unknown. Although the participation of shelterin proteins in the regulation of telomerase function has been shown to some extent [75], the specifics of their interplay in lung tissue, and especially in the process of its fibrotic restructuring, remain to be revealed. In particular, Tin2 is a known telomerase recruitment factor [76], and is known to be depleted in the fibrotic lung [31], yet the effect of this depletion on telomerase activity needs further study.

To what extent the disruptions in function of CST complex observed under other pathological conditions [30] may apply to the cellular populations of the fibrotic lung is as yet an open question, despite some data regarding connections between variants of STN1 (also called OBFC1) and the pathology of sporadic IPF [77]. Moreover, Coats plus syndrome, a pathology involving STN1 mutation, has been reported to affect fibroblast populations of the patient; fibroblasts were reported to be abnormally large and vacuolated, exhibiting proliferative arrest after a few divisions [78]. However, despite indications that loss of STN1 function leads to cellular senescence [79], no increase in SA-β-Gal staining (indicative of senescence) was reported for fibroblasts in the cases of Coats plus syndrome [78], and no pulmonary symptoms are mentioned in association with the pathology. This raises the question of a possible mechanism for suppression of lung fibrosis in the cases of Coats plus syndrome.

Cajal bodies are known to process telomerase RNA [80] and promote telomerase association with telomeric DNA [81]. Their importance to cell proliferation makes them a valuable target for anti-proliferative therapies [82], which would indicate a good prospective for the treatment of ILDs. Nonetheless, their role in fibroblast proliferation during pulmonary fibrosis remains virtually unstudied, thus making them one of the most promising topics of future research on the role of telomere maintenance in IPF. In this regard, it is important to admit the lack of studies linking abnormalities of dyskerin function to interstitial lung pneumonia. Dyskerin is known to be an active component of telomerase complex [83], and Cajal body coilin and SMN proteins have been demonstrated to regulate telomerase activity through blocking of dyskerin association with telomerase RNA [84]. Moreover, localization of *DKC1* (dyskerin gene) at the X chromosome raises the possibility of the impact of its mutations on the phenotype, including hereditary pathologies such as familial interstitial pneumonia. Nevertheless, a study by Kropski et al. [59] remains virtually the only research directly linking dyskerin mutation to a case of ILD. The subject of DKC1 product’s involvement in fibrosis, and lung fibrosis in particular, requires further investigation.

ATM (ataxia-telangiectasia mutated) protein is a dsDNA break response factor that activates a multitude of signal cascades and is known to participate in telomere maintenance via telomerase activation [85]. Although it has been shown to be activated in pathological conditions, including IPF [86,87], the details of its part in fibroblast survival and proliferation under ILDs are also lacking at present. It can be surmised that oxidative stress, which frequently accompanies pulmonary fibrosis, is one of the causes of the observed upregulation of ATM; however, the differences of this process in epithelial and mesenchymal cell populations of the diseased lung still need to be revealed. T-loop (telomere end-loop) is one of the factors involved in ATM regulation; namely, its formation (involving TRF2 homodimer binding) suppresses ATM expression, while linearization of telomeric DNA leads to ATM activation [88]. TRF2, in turn, is a known protooncogenic factor actively expressed in proliferating cells [31], which may also be relevant to myofibroblast accumulation during fibrosis. Therefore, structural studies may help reveal the details of ATM regulation in pulmonary fibrosis.

Telomere maintenance and telomeric DNA linearization are intricately tied to cell cycle regulation and cell cycle arrest [89], which is an important consideration, as variations in the cell cycle are characteristic of the progression of classic pulmonary fibrosis [90]. Moreover, cell cycle arrest and cell senescence are directly dependent on telomere length maintenance [91]. Since it is known that cell senescence of myofibroblasts plays an essential role in the progression of pulmonary fibrosis [92], it makes the topic of telomere maintenance in various cell lines involved in the progression of ILDs all the more crucial.

## 6. Summary and Unsolved Problems

Idiopathic pulmonary fibrosis is one of the most common manifestations of a telomere-related disease (ILDs). Genetic mutations related to telomere maintenance are one of many genetic defects found in patients with familial IPF. Mutations in telomerase genes and shortened telomeres influence the course of the disease and tend to have a worse prognosis and more extrapulmonary manifestations.

Taken together, it is important to identify IPF patients early, to introduce antifibrotic drugs and refer them to a lung transplant center. However, and unfortunately, there are neither good and available screening tools nor efficient treatments for these patients in clinical practice.

The evaluation of telomere length is not included in the diagnostic algorithm of IPF or other fibrotic diseases due to the limited evidence about the utility of this test. Furthermore, telomere length testing is only available through a minority of expert centers for interstitial lung disease. However, identification the clinical signs that increase the probability of telomere shortening is possible and could help in diagnostic and treatment decisions. Many questions remain open regarding screening and treatment, such as: Who should be screened, and what tests should be performed in suspected genetically determined IPF (e.g., genetic analysis of telomerase-related genes, telomere length measurement or both)? What is the best treatment for IPF patients? Could Nintedanib be more efficient that pirfenidone, giving its anti-cancer role? Should danazol be added to anti-fibrotic therapy or as supplementary therapy after lung transplantation in these patients?

In summary, further research is needed in order to better understand how to manage IPF patients before and after lung transplantation, and to try to tailor personalized medicine.

## Figures and Tables

**Table 1:** Association between telomere-related mutation and interstitial lung disease.

Telomere-Related Mutation	Interstitial Lung Disease ^1^	References
*Tert*	IPF; NSIP; AIP; COP; SR-ILD; HP; PPFE	[46,48,58,59]
*Tert*	IPF; NSIP; PPFE; HP	[46,48,59]
*PARN*	IPF; NSIP; HP	[43,59]
*TINIF2*	IPF	[60]
*NAF1*	IPF	[61]
*DKC1*	IPF; NSIP	[59]
*RTEL1*	IPF; NSIP; PPEF; HP	[43,48]

^1^ IPF: idiopathic pulmonary fibrosis; NSIP: nonspecific intestinal pneumonitis, AIP: acute intestinal pneumonia; COP: cryptogenic organizing pneumonia; SR-ILD: smoking related interstitial lung disease; HP: hypersensitivity pneumonitis; PPFE: pleuroparenchymal fibroelastosis.

## Abbreviations

ILD	Interstitial lung disease
IPF	Idiopathic pulmonary fibrosis
UIP	Usual interstitial pneumonia
FPF	Familial pulmonary fibrosis
lncRNA	Long non-coding RNA
NSIP	Nonspecific intestinal pneumonitis
